# Immunotherapy Responses in Viral Hepatitis-Induced HCC: A Systematic Review and Meta-Analysis

**DOI:** 10.3390/curroncol31110532

**Published:** 2024-11-15

**Authors:** Junaid Anwar, Hafiz Muhammad Arslan, Zouina Sarfraz, Juwairiya Shuroog, Ahmed Abdelhakeem, Ali Saeed, Anwaar Saeed

**Affiliations:** 1 Department of Medicine, Baptist Hospitals of Southeast Texas, Beaumont, TX 77701, USA; jxa205@shsu.edu; 2 Department of Medicine, Lincoln Medical and Mental Health Center, Bronx, NY 10451, USA; hafiza1@nychhc.org; 3 Department of Medicine, Fatima Jinnah Medical University, Lahore 54000, Pakistan; zouina.sarfraz@fjmu.edu.pk; 4 Department of Medicine, TidalHealth Peninsula Regional, Salisbury, MD 21801, USA; juwairiya.shuroog@tidalhealth.org; 5 Department of Medicine, Division of Hematology & Oncology, Mayo Clinic College of Medicine and Science, Jacksonville, FL 32224, USA; abdelhakeem@shsu.edu; 6 Department of Medicine, Ochsner Lafayette General Medical Center, Lafayette, LA 70503, USA; asaeedmd@gmail.com; 7 Department of Medicine, Division of Hematology & Oncology, University of Pittsburgh Medical Center, Pittsburgh, PA 15232, USA

**Keywords:** hepatocellular carcinoma, immunotherapy, hepatitis B, hepatitis C, objective response, survival, clinical trials, cancer treatment, personalized medicine

## Abstract

**Background**: Hepatocellular carcinoma (HCC) is a prevalent liver cancer with poor prognosis, often linked to hepatitis B (HBV) and C (HCV) infections. This meta-analysis evaluates the efficacy of immunotherapy in HCC, particularly in cases arising from viral hepatitis. **Methods**: In adherence to PRISMA Statement 2020 guidelines, the immunotherapeutic outcomes comprised objective response rate (ORR), progression-free survival (PFS), and overall survival (OS). Data were analyzed from randomized controlled trials up to April 2024 using the fixed-effects models in R (V.4.3.3.) and RevMan (Cochrane). **Results**: This study included 9 trials with 5316 patients. The ORR was slightly higher in the viral group at 27.93% compared to 24.07% in the non-viral group, though this difference was not significant (*p* = 0.15). Viral HCC patients exhibited a median PFS of 7.3 months (IQR: 6.2–8.4) compared to 5.8 months (IQR: 5.48–6.13) in non-viral patients, a significant improvement (*p* = 0.005). Similarly, median OS was longer in the viral group at 16.8 months (IQR: 12.99–20.61) versus 15.2 months (IQR: 13.25–17.15) for non-viral HCC, which was also significant (*p* < 0.0001). The median OS for viral HCC was 16.8 months (IQR: 14.11–19.49 months), with HBV patients experiencing slightly higher survival at 17.15 months (IQR: 14.3–20 months) compared to 16.8 months (IQR: 12.99–20.61 months) for HCV patients; this difference was not statistically significant (*p* = 0.89). **Conclusions**: Immunotherapy shows potential in treating HCC, with significantly better outcomes in viral HCC, particularly HBV-associated cases. The heterogeneity highlights the need for personalized treatment approaches based on the viral background of HCC patients. Further research should aim to optimize these therapies to improve survival rates.

## 1. Introduction

Hepatocellular carcinoma (HCC), a common form of liver tumor, represents over 90% of the liver’s primary tumors [1,2]. Globally, HCC stands as the fifth leading cancer type, with around 841,000 individuals diagnosed in 2018 [3]. This condition is predominantly found in males, with a rate three times higher than in females, and the median diagnosis age in the United States is 64 [4,5]. Nearly 780,000 deaths in 2018 were attributed to HCC, placing it as the second most lethal cancer among men, following only lung cancer [6]. The prevalence of HBV and, consequently, the incidence of HBV-related HCC exhibit notable geographic disparities due to variations in HBV incidence across the world. The Asian-Pacific region and sub-Saharan Africa are identified as having the highest rates of HCC globally [7]. In contrast, the United States experiences a markedly lower incidence of HBV-associated HCC, with it accounting for less than 20% of HCC cases [8].

Cirrhosis, often a consequence of chronic liver disease, is present in about 85% of those diagnosed with HCC, making it a critical risk factor [9]. Factors leading to liver cancer and subsequent deaths globally are majorly hepatitis B (HBV) (33%), alcohol consumption (30%), hepatitis C (HCV) (21%), and other causes (16%) [10,11]. Notably, the majority of HCC diagnoses are in individuals with a history of hepatitis, especially those born between 1945 and 1965, indicating a strong correlation with HCV infection [5,12].

Despite being common, the prognosis for HCC remains poor, with a five-year survival rate of just 18%, which is only marginally better than that of pancreatic cancer [13,14]. This low survival rate highlights the aggressive nature of HCC and highlights the urgent need for effective treatment strategies. The high incidence of HCC in regions burdened with hepatitis B emphasizes the global challenge of combating this cancer, especially in settings where medical resources may be limited [15,16].

Globally, chronic HBV and HCV infections are recognized as the leading risk factors for developing HCC, contributing to over half of the cases worldwide [17,18]. Despite a global decrease in HBV cases following the widespread adoption of HBV vaccinations, the incidence of HBV remains significantly high in certain regions [19,20]. Currently, there are an estimated 350 to 400 million individuals worldwide living with HBV, predominantly in Asia or of Asian origin [21,22]. HBV is responsible for approximately 749,000 new HCC cases and 692,000 deaths related to HCC annually [23]. The yearly risk of developing HCC is less than 1% for HBV-infected individuals without cirrhosis and increases to 2–3% among those with cirrhosis [24,25].

Treatment options for early-stage HCC include surgical resection, liver transplantation, and local ablative therapies like microwave ablation (MWA) and radiofrequency ablation (RFA) [26]. For those with advanced stages of HCC, systemic therapies such as targeted therapy and chemotherapy are suggested [27]. However, HCC shows limited responsiveness to conventional systemic chemotherapy, a challenge often attributed to the presence of the multidrug resistance gene protein on cancer cell surfaces, which actively pumps out chemotherapeutic agents [28].

Immunotherapy, which involves modulating the immune system either to enhance or suppress its activity, is utilized across a spectrum of immunological disorders, including but not limited to immunodeficiencies, autoimmune diseases, hypersensitivities, and various forms of cancer, as well as in the context of organ transplants, inflammatory conditions, and infectious diseases [29]. This approach aims to improve patients’ quality of life and life expectancy by utilizing the body’s immune response. The immune system plays a pivotal role in the pathogenesis of HCC. The ability of dendritic cells to present antigens decreases, impairing the activation of CD8+ T-cells (CTLs). Viral components trigger the activation of M1 macrophages; however, because of immune dysregulation, an alternative macrophage phenotype, M2, becomes activated, which further hinders the function of CTLs [30]. In finding curative treatments for cancer, including HCC, the U.S. Food and Drug Administration (FDA) has approved seven immune checkpoint inhibitors (ICIs) targeting proteins such as programmed death protein-1 (PD-1), cytotoxic T lymphocyte-associated antigen 4 (CTLA-4), and programmed death-ligand 1 (PD-L1) [31,32]. Additionally, emerging immunotherapeutic strategies, including the use of chimeric antigen receptor-modified immune cells, adoptive cell therapies, cytokines, and cancer vaccines, offer hope for HCC patients, nearing readiness for broader application [33].

This meta-analysis aims to synthesize existing research to evaluate the efficacy and potential of immunotherapy in treating HCC, particularly in the backdrop of viral hepatitis infections, where options are limited, and the prognosis is poor. Given the minimal responsiveness of viral hepatitis-induced HCC to systemic chemotherapy and the promise of immunotherapeutic strategies, it is crucial to assess the effectiveness of such treatments. By meta-analyzing the outcomes of various immunotherapeutic approaches, this study aims to enhance patient care and survival rates.

## 2. Materials and Methods

### 2.1. Study Design and Registration

This meta-analysis was conducted following the Preferred Reporting Items for Systematic Reviews and Meta-Analyses (PRISMA) 2020 guidelines to ensure rigorous and transparent reporting of its findings.

### 2.2. Search Strategy and Study Selection

An exhaustive literature search was performed across multiple databases, including PubMed, Embase, and the Cochrane Central Register of Controlled Trials (CENTRAL), up to 5 April 2024. The search strategy was designed to obtain all relevant studies, employing a combination of keywords and medical subject headings (MeSH) related to “Hepatocellular Carcinoma”, “Immunotherapy”, “Viral Hepatitis”, “PD-1”, “PD-L1”, “CTLA-4”, and specific trial names and identifiers as necessary. The search was unconstrained by language or publication date to maximize inclusivity.

**Figure 1:**
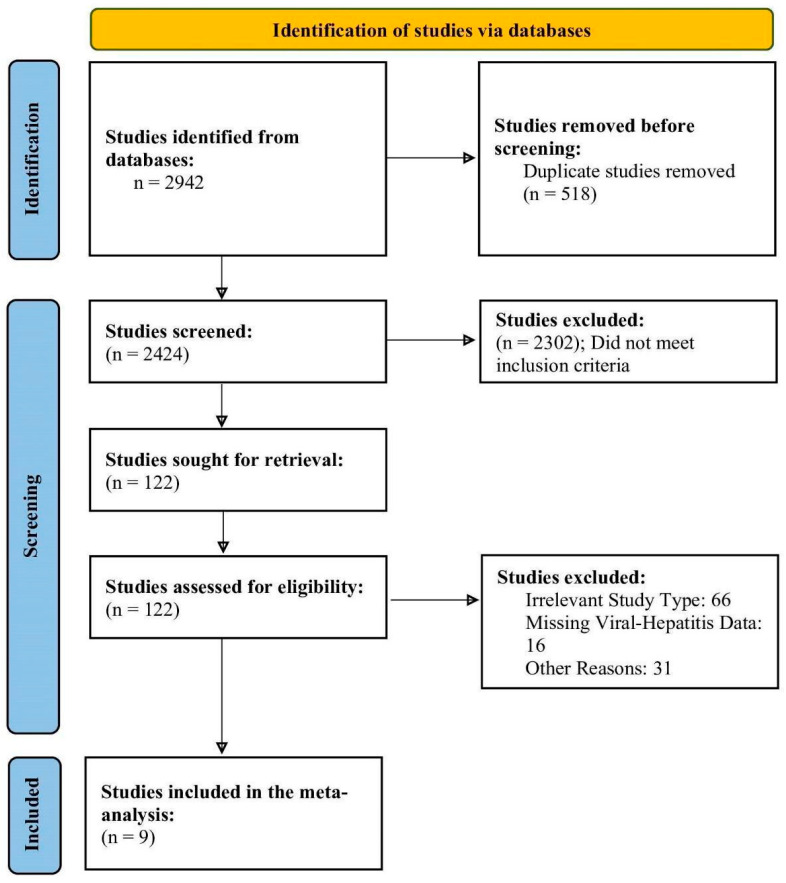
PRISMA flowchart depicting the study selection process.

Of 2942 studies identified from the databases, 2424 studies were screened post-duplicate removal. Of those, only 122 studies were sought for full-text eligibility, as the excluded studies (n = 2302) did not meet the eligibility criteria on title/abstract screening. Therefore, a total of 9 trials were included in this meta-analysis (Figure 1).

### 2.3. Eligibility and Inclusion/Exclusion Criteria

Studies were eligible for inclusion only if they were randomized controlled trials (RCTs) involving patients diagnosed with viral hepatitis-induced HCC. Eligible studies needed to have reported on the efficacy of immunotherapy treatments, focusing on outcomes such as objective response rate (ORR), progression-free survival (PFS), and overall survival (OS). Studies were excluded if they did not differentiate outcomes based on viral etiology or if they involved non-immunotherapy interventions. The included trials investigated immunotherapy treatments that target PD-1 or PD-L1 receptors, such as camrelizumab, atezolizumab, nivolumab, and durvalumab. Immunotherapy was given alone or in combination with tyrosine kinase inhibitors. Most trials included locally advanced or metastatic and/or unresectable HCC. Other key inclusion criteria included patients 18 years of age or older with histologically confirmed hepatocellular carcinoma who had no prior systemic therapy and were ineligible for locoregional therapy; patients who had Barcelona Clinic Liver Cancer stage B or C, Child–Pugh Score class A, Eastern Cooperative Oncology Group (ECOG) performance status score of 0 or 1, and at least one measurable lesion per Response Evaluation Criteria in Solid Tumors, version 1.1 (RECIST v1.1); patients with hepatitis B virus infection, who had a viral load of hepatitis B virus DNA lower than 500 IU/mL or less than 2500 copies per mL; and patients testing positive for hepatitis C virus RNA who had hepatic function meeting the eligibility criteria could also be enrolled if they agreed to receive anti-viral therapy per local standard of care throughout the study. Patients were ineligible if they had fibrolamellar or sarcomatoid hepatocellular carcinoma, mixed cholangiocarcinoma and hepatocellular carcinoma, previous liver transplant, history of hepatic encephalopathy, clinically significant ascites, portal hypertension with bleeding, esophageal or gastric varices within the past 6 months, or active brain metastases.

### 2.4. Data Extraction and Management

Data were extracted systematically by two independent reviewers using a standardized form. Discrepancies were resolved through consensus or consultation with a third reviewer. Extracted information included study characteristics (author, year, phase, sample size), hepatitis status, details of the immunotherapy interventions (drugs, dosages), and key findings (ORR, PFS, OS).

### 2.5. Statistical Analysis

Pooled estimates of ORR, PFS, and OS were calculated using fixed-effects models. Heterogeneity was quantified using the I^2^ statistic, with values >50% indicating significant heterogeneity. Subgroup analyses were conducted, where possible, based on viral etiology (HBV vs. HCV) and type of immunotherapy intervention.

A leave-one-out meta-analysis was conducted to evaluate the robustness of the meta-analytic estimates for outcomes where the I^2^ statistic had values greater than 50%. This analysis was carried out by sequentially excluding each study from the meta-analysis and recalculating the pooled estimates using fixed-effects models. This approach allowed us to assess the influence of individual studies on the overall meta-analytic results, ensuring that no single study disproportionately affected the outcomes.

*p*-values were computed with significance at or below 0.05. All statistical analyses were carried out using the software R 4.1.3 using the “meta” package and Review Manager 5.4.3 (Cochrane), which are specifically designed for conducting meta-analyses.

### 2.6. Risk of Bias Assessment

The evaluation of the risk of bias for individual studies included in this meta-analysis was conducted using the updated RoB 2 tool for randomized trials developed by the Cochrane Collaboration. This tool provides a structured approach to assessing biases related to the randomization process, deviations from intended interventions, missing outcome data, measurement of the outcome, and selection of the reported result. Each study was critically appraised across these specified domains, ensuring a thorough examination of potential biases that could influence the reliability and validity of the study findings. Discrepancies in the risk of bias assessments were resolved through discussion or by involving a third reviewer, ensuring a consensus-based approach to risk determination.

## 3. Results

### 3.1. Systematic Review Findings

A total of 5316 patients were pooled across 9 trials. The characteristics of the included studies are listed in Table 1. Included in this review are phase 2 trials such as KEYNOTE-224 (2018) and CheckMate 040 (2020) and phase 3 trials including RATIONALE-301, CARES-310, HIMALAYA, IMbrave 150, COSMIC-312, and CheckMate-459, spanning from 2022 to 2023. There is a noticeable progression to phase 3 trials, indicating a move to validate the efficacy and safety of therapies on a larger scale. The integration of ICIs targeting PD-1/PD-L1 and CTLA-4 pathways in numerous trials signals a significant shift towards using the immune system against HCC, diverging from traditional treatment methods. The exploration of combination therapies, particularly in COSMIC-312 and IMbrave 150, reflects their potential to provide superior outcomes. The median follow-up duration for the included trials is 13.9 months (IQR = 16.3).

The RATIONALE-301 trial compares tislelizumab, an anti-PD-1 antibody administered at 200 mg intravenously every 3 weeks, with sorafenib, highlighting the potential of PD-1 inhibitors in HCC management. Similarly, the CARES-310 trial investigates the combination of camrelizumab, a PD-L1 inhibitor given at 200 mg every 2 weeks, with rivoceranib, a VEGFR inhibitor taken at 250 mg orally four times daily, suggesting a synergistic approach to disrupt tumor microenvironments. In the HIMALAYA trial, a novel dual-immunotherapy strategy employing a single dose of tremelimumab (300 mg) followed by durvalumab (1500 mg every 4 weeks) was explored, indicating the enhanced immunogenic response achieved by combining CTLA-4 and PD-L1 inhibitors. The IMbrave 150 trial further validates the benefit of integrating PD-L1 blockade with anti-angiogenesis, through the administration of atezolizumab (1200 mg) with bevacizumab (15 mg/kg) intravenously every 3 weeks, showcasing significant advancements in HCC management. The COSMIC-312 trial presents a regimen of cabozantinib (40 mg orally once daily) plus atezolizumab (1200 mg intravenously every 3 weeks), exploring the efficacy of PD-L1 inhibition combined with tyrosine kinase inhibition. Meanwhile, the CheckMate-459 trial focuses on the viability of sole PD-1 inhibition in HCC treatment by comparing nivolumab, administered at 240 mg intravenously every 2 weeks, against sorafenib. ORIENT-32 combines sintilimab (200 mg IV every 3 weeks) with IBI305 (15 mg/kg every 3 weeks), a bevacizumab biosimilar, versus sorafenib, exploring the effectiveness of PD-1 inhibition alongside angiogenesis inhibition. CheckMate 040 diversifies the approach by offering varied regimens of nivolumab, including monotherapy and combination with ipilimumab, to explore the additional benefits of CTLA-4 co-inhibition. The KEYNOTE-224 trial assesses pembrolizumab, given at 200 mg intravenously every 3 weeks for up to 2 years, further contributing to the evidence supporting the role of immunotherapy, particularly PD-1 blockade, in HCC management (Table 1).

### 3.2. Meta-Analytical Findings

#### 3.2.1. Objective Response Rate

**Figure 2:**
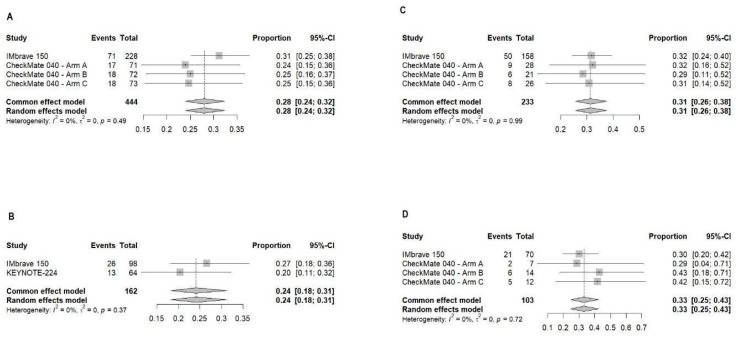
Forest plot of ORR with ICI use in hepatitis-induced showing a consistent significant effect across the viral group studies and no comparable significant difference from the non-viral group (**A**) and non-viral groups (**B**). HBV (**C**) and HCV-only (**D**) outcomes showing comparable treatment efficacy are also depicted [37,41,42].

For the viral group, our meta-analysis encompassed two trials with a cumulative total of 444 observations and identified 124 events (treatment responses) (Figure 2A). In the IMbrave 150 study, the response proportion was observed at 31.14% (95% CI: 25.19% to 37.59%) indicating efficacy within this cohort. The CheckMate 040 trial was segmented into three distinct arms. Arm A of the CheckMate 040 study reported a response proportion of 23.94% (95% CI: 14.61% to 35.54%). Arm B followed closely, with a response proportion of 25% (95% CI: 15.54% to 36.6%). Arm C showed a response proportion of 24.66% (95% CI: 15.32% to 36.14%). The analysis demonstrated no heterogeneity among the studies (τ^2^ = 0; I^2^ = 0%), suggesting a consistent effect across the viral group studies.

The non-viral group’s analysis incorporated data from two trials, with a total of 162 observations and 39 events. The individual and pooled outcomes were 26.53% (95% CI: 18.12% to 36.41%) for IMbrave 150. They were 20.31% (95% CI: 11.28% to 32.23%) in KEYNOTE-224. The pooled ORR for the non-viral group, similar to the viral group, was estimated at 24.07% [18.11%; 31.25%], with the analysis also showing no evidence of heterogeneity (τ^2^ = 0; I^2^ = 0%) (Figure 2B).

The absence of heterogeneity in both groups (I^2^ = 0%) highlights a consistent treatment effect across the studies analyzed. The similarity in the pooled ORR between the viral (27.93%) and non-viral (24.07%) groups, along with overlapping confidence intervals, suggests comparable efficacy of HCC treatments regardless of etiological factors.

We specifically analyzed the ORR for HBV and HCV infected populations. The analysis included data from the IMbrave 150 trial and CheckMate 040 (across three arms), allowing for a comparative assessment of treatment responses in these viral groups.

For the HBV-infected cohort, the meta-analysis incorporated two trials with a total of 233 observations and 73 events (Figure 2C). The individual study ORRs, along with their 95% CIs, were 31.65% [24.49%; 39.51%] for IMbrave 150, 32.14% [15.88%; 52.35%] for CheckMate 040—Arm A, 28.57% [11.28%; 52.18%] for CheckMate 040—Arm B, and 30.77% [14.33%; 51.79%] for CheckMate 040—Arm C. There was no observed heterogeneity among the studies (τ^2^ = 0; I^2^ = 0%).

The HCV group’s meta-analysis also spanned two trials but with a smaller cohort of 103 observations and 34 events (Figure 2D). The individual ORRs and their 95% CIs were 30% [19.62%; 42.13%] for IMbrave 150, 28.57% [3.67%; 70.96%] for CheckMate 040—Arm A, 42.86% [17.66%; 71.14%] for CheckMate 040—Arm B, and 41.67% [15.17%; 72.33%] for CheckMate 040—Arm C.

The analyses of HBV and HCV groups demonstrated comparable treatment efficacy with pooled ORRs of 31.33% and 33.01%, respectively. Notably, the absence of statistical heterogeneity within each viral group (I^2^ = 0%) highlights the consistency of treatment response across the analyzed studies.

#### 3.2.2. Progression-Free Survival

The median PFS for both HBV and HCV was 7.3 months (Interquartile Range [IQR]: 6.2–8.4 months). Specifically, the median PFS was 6.7 months (IQR: 5–8.4 months) for HBV-infected patients and 8.35 months (IQR: 5.18–11.53 months) for HCV-infected patients, demonstrating a better PFS outcome in the HCV group compared to the HBV group. Non-viral HCC patients showed a median PFS of 5.8 months (IQR: 5.48–6.13 months), which was lower than that observed in patients with viral hepatitis.

The meta-analysis on PFS (in months) rates across viral and non-viral groups in patients with HCC integrates outcomes from two trials: IMbrave 150 and COSMIC-312.

**Figure 3:**
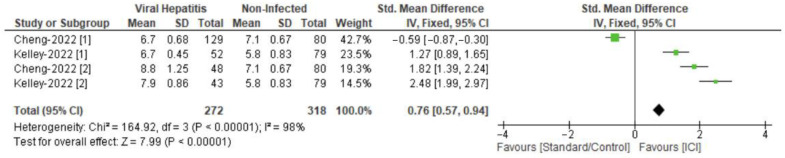
Forest plot of PFS with ICI use in hepatitis-induced and non-viral groups, indicating a statistically significant favorable effect of ICIs on PFS in the overall HCC patient population (effect size computations are depicted) [37,38].

The analysis included a total of 272 patients with hepatitis-induced HCC and 159 patients with non-infectious HCC. Cohen’s d was 0.76, with a confidence interval ranging from 0.57 to 0.94. This indicates a statistically significant favorable effect of ICIs on PFS in the overall HCC patient population. The test for overall effect was highly significant (Z = 7.99, *p* < 0.00001), confirming the beneficial impact of the interventions on PFS. There was considerable heterogeneity among the studies (Chi^2^ = 164.92, df = 3, *p* < 0.00001), with an I^2^ of 98% (Figure 3).

In the leave-one-out meta-analysis examining PFS rates in HCC trials, significant effects were noted when different arms were excluded from the analyses.

Starting with the IMbrave 150 trial, excluding the first arm revealed a considerable increase in PFS, showing an effect size of 1.76 with a 95% confidence interval of 1.52 to 2.01. This analysis presented high heterogeneity (I^2^ = 86%, *p* = 0.0006) and a notably significant overall effect (*p* < 0.00001). Conversely, removing the second arm led to a reduced effect size of 0.51, with a 95% confidence interval from 0.30 to 0.71, alongside extremely high heterogeneity (I^2^ = 99%, *p* < 0.00001) and a significant test for overall effect (*p* < 0.00001).

Similarly, in the COSMIC-312 trial, the exclusion of the first arm resulted in a moderate effect size of 0.60, with a 95% confidence interval of 0.39 to 0.81, and very high heterogeneity (I^2^ = 99%, *p* < 0.00001) paired with a significant overall effect (*p* < 0.00001). Excluding the second arm yielded an effect size of 0.47, with a 95% confidence interval of 0.26 to 0.67, high heterogeneity (I^2^ = 98%, *p* < 0.00001), and a notable overall effect (*p* < 0.00001).

**Figure 4:**
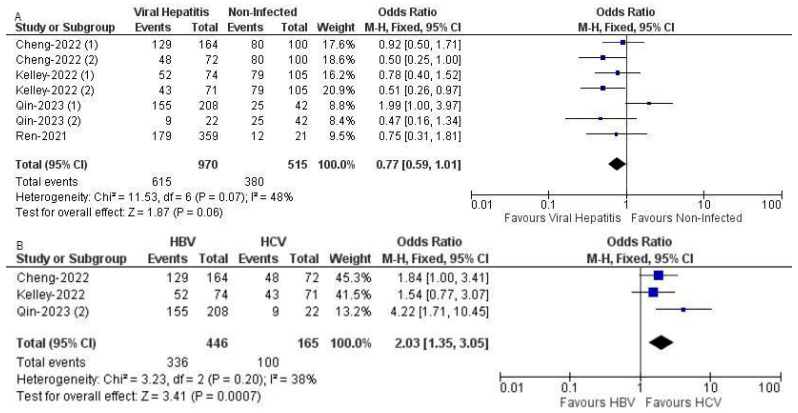
(**A**) Forest plot of PFS in hepatitis-induced and non-viral groups with ICI use across 6 trials with 13 arms with data on proportional survival outcomes, indicating a non-significant effect but a trend towards better PFS in the viral group [34,35,37,38,40]. (**B**) Forest plot of OS in HBV vs. HCV groups with ICI use across five trials and six total arms with data on proportional survival outcomes, showing significant difference favoring HBV patients [35,37,38].

The meta-analysis of proportional PFS outcomes included data from four trials: CARES-310, IMbrave 150, COSMIC-312, and ORIENT-32, spanning a total of seven arms. The analysis encompassed 970 individuals in the viral hepatitis group and 515 in the non-infected group. The median follow-up duration was 10 months (IQR: 2). The odds ratio for PFS was 0.77 with a 95% CI of 0.59 to 1.01, indicating a trend towards better PFS in the viral hepatitis group compared to the non-infected group, though this finding was not statistically significant (*p* = 0.06). The total number of progression events was 615 in the viral hepatitis group compared to 380 in the non-infected group. The heterogeneity among the included studies was moderate, evidenced by an I^2^ value of 48% and a chi-squared statistic of 11.53 on 6 degrees of freedom (*p* = 0.07), suggesting some variability but reasonable consistency among the studies (Figure 4).

In a separate analysis focusing on PFS outcomes between HBV and HCV patients, data from three trials—CARES-310, IMbrave 150, and COSMIC-312—were analyzed, involving 446 patients in the HBV group and 165 in the HCV group. The median follow-up duration was 10 months (IQR: 4). This analysis revealed a significant difference in PFS, with a combined odds ratio of 2.03 (95% CI: 1.35, 3.05), favoring HBV patients over HCV patients (*p* = 0.0007). There were 336 total progression events in the HBV group and 100 in the HCV group. The heterogeneity in this subset was relatively low, with an I^2^ of 38% and a chi-squared statistic of 3.23 on 2 degrees of freedom (*p* = 0.20), indicating less variability in the PFS outcomes between the groups compared to the broader analysis.

#### 3.2.3. Overall Survival

**Figure 5:**
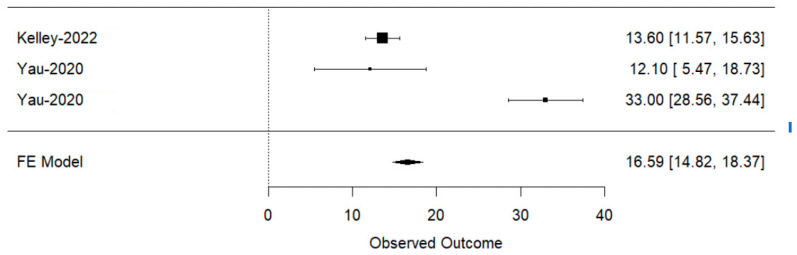
Forest plot of OS in hepatitis-induced and non-viral groups with ICI use across three arms with reported data on SD [38,39]. Kelley-2022 arm 2 refers to COSMIC-312’s second arm. Yao-2020, B1 and C2 arms refers to two arms of CheckMate 040.

Regarding overall survival, the median OS for both HBV and HCV patients was 16.8 months (IQR: 14.11–19.49 months). The median OS was slightly higher in HBV-infected patients at 17.15 months (IQR: 14.3–20 months) as compared to 16.8 months (IQR: 12.99–20.61 months) in HCV-infected patients. The non-viral group had a median OS of 15.2 months (IQR: 13.25–17.15 months), indicating a slightly lower survival rate compared to the viral hepatitis groups. On meta-analyzing the OS (mean data reported in months), the results of the fixed-effects model showed an estimated effect size of 16.59 with a standard error of 0.9059. The Z-value for this estimate was 18.3147, resulting in a highly significant *p*-value of less than 0.0001. The 95% confidence interval for the effect size ranged from 14.8155 to 18.3665. A test for heterogeneity gave a Q-statistic of 62.5462 with 2 degrees of freedom, and the associated *p*-value was less than 0.0001, confirming significant heterogeneity within the analyzed studies (Figure 5).

In the leave-one-out meta-analysis concerning OS rates across viral and non-viral groups in patients with HCC, each trial’s study arm was sequentially excluded to assess its influence on the meta-analytic estimates.

When the “Kelley-2022” arm, representing COSMIC-312’s second arm, was excluded, the estimated effect on OS was markedly high at 26.53, with a confidence interval ranging from 22.84 to 30.22, a *p*-value of less than 0.0001, and a very high heterogeneity (I^2^ = 96.21%). This indicates a substantial reduction in the overall effect size when this arm is removed, suggesting that it contributes significantly to enhancing the OS rates observed in the combined data.

Excluding the “Yau-2020-B1” arm, which is one of the arms of CheckMate 040, resulted in a moderate effect size of 16.95, with a confidence interval from 15.11 to 18.80, a *p*-value less than 0.0001, and extremely high heterogeneity (I^2^ = 98.35%). This outcome demonstrates that the first arm of CheckMate 040 also plays a critical role in the pooled analysis but has a lesser impact compared to the “Kelley-2022” arm.

Finally, removing the “Yau-2020-C2” arm, the second arm of CheckMate 040, yielded the lowest effect size of 13.47, with a confidence interval between 11.53 and 15.41, a *p*-value less than 0.0001, and an I^2^ of 0%. This result highlights that this arm has the least influence on the overall OS rates in the meta-analysis, suggesting a more modest contribution to the pooled outcome.

**Figure 6:**
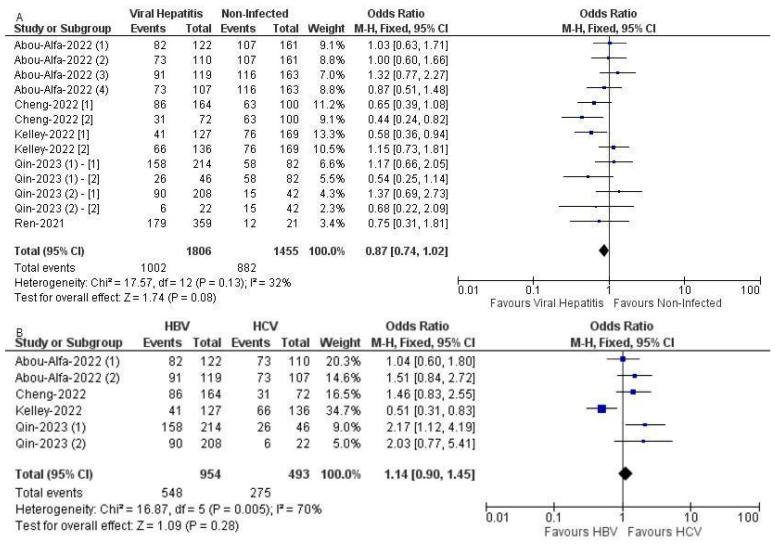
(**A**): Forest plot of OS in hepatitis-induced and non-viral groups with ICI use across 6 trials with 13 arms with data on proportional survival outcomes, indicating a non-significant trend towards better survival in the hepatitis-induced group compared to the non-viral group [34,35,36,37,38,40]. (**B**): Forest plot of OS in HBV vs. HCV groups with ICI use across five trials and six total arms with data on proportional survival outcomes indicating a non-significant difference [34,35,36,37,38].

This meta-analysis of proportional survival outcomes included data from six trials: RATIONALE-301, CARES-310, HIMALAYA trial, IMbrave 150, COSMIC-312, and ORIENT-32, incorporating a total of 13 arms. The analysis included 1806 individuals in the hepatitis-induced group and 1455 in the non-viral group. The median follow-up duration for the included trials in this analytical subset was 13.9 months (IQR: 20.93). The pooled odds ratio for OS was 0.87 with a 95% CI of 0.74 to 1.02, indicating a non-significant trend towards better survival in the hepatitis-induced group compared to the non-viral group, though this did not reach statistical significance (*p* = 0.08). The total number of events (deaths) was 1002 in the hepatitis-induced group and 882 in the non-viral group. The heterogeneity among the studies was moderate, with an I^2^ value of 32% and a chi-squared statistic of 17.57 on 12 degrees of freedom (*p* = 0.13), suggesting reasonable consistency among the included studies (Figure 6A).

This analysis of proportional survival outcomes of ICIs in patients with HBV compared to HCV encompassed data from five trials: RATIONALE-301, CARES-310, HIMALAYA trial, IMbrave 150, and COSMIC-312. This study involved 954 patients in the HBV group and 493 in the HCV group. The median follow-up duration for the included trials in this analytical subset was 14.5 months (IQR: 24.2). The combined odds ratio was 1.14 with a 95% CI from 0.90 to 1.45, which did not indicate a significant difference in survival between HBV and HCV patients (*p* = 0.28). There were 548 total events in the HBV group and 275 in the HCV group. Notably, the heterogeneity in this analysis was high, with an I^2^ of 70% and a chi-squared statistic of 16.87 on 5 degrees of freedom (*p* = 0.005), indicating significant variability in the OS outcomes between the trials involving HBV and HCV patients (Figure 6B).

The leave-one-out meta-analysis on OS rates HBV and HCV groups involved removing each study one at a time and analyzing the impact on the overall results. This analysis highlights the variance in outcomes influenced by each individual trial:

- Excluding the first arm of the HIMALAYA trial yielded an odds ratio of 1.17 (95% CI: 0.89, 1.53) with a moderate heterogeneity of 76% (Chi^2^ = 16.71, *p* = 0.002) and no significant overall effect (*p* = 0.25).
- Removing the second arm of the HIMALAYA trial resulted in a slightly lower odds ratio of 1.08 (95% CI: 0.83, 1.41), with similar heterogeneity (75%, Chi^2^ = 15.86, *p* = 0.003) and an insignificant overall effect (*p* = 0.57).
- Omitting IMbrave 150 from the analysis also presented an odds ratio of 1.08 (95% CI: 0.83, 1.41), with heterogeneity remaining high at 75% (Chi^2^ = 16.02, *p* = 0.003) and no significant impact on overall results (*p* = 0.57).
- Excluding COSMIC-312 brought about a noticeable increase in the odds ratio to 1.48 (95% CI: 1.12, 1.96), significantly reducing heterogeneity to 0% (Chi^2^ = 3.32, *p* = 0.51) and showing a significant overall effect (*p* = 0.006).
- Leaving out RATIONALE-301 resulted in an odds ratio of 1.04 (95% CI: 0.80, 1.35), with substantial heterogeneity (69%, Chi^2^ = 12.73, *p* = 0.01) and a non-significant overall effect (*p* = 0.76).
- Removing CARES-310 yielded an odds ratio of 1.10 (95% CI: 0.85, 1.41), with high heterogeneity (74%, Chi^2^ = 15.49, *p* = 0.004) and an insignificant effect on overall results (*p* = 0.47).

This sensitivity analysis revealed how each trial contributed differently to the pooled estimates, with COSMIC-312 notably enhancing the PFS ratio and reducing heterogeneity to 0% when removed. In contrast, other trials, such as RATIONALE-301 and CARES-310, showed minimal influence on the meta-analytic conclusions, suggesting varying levels of impact from different studies on the overall findings.

**Figure 7:**
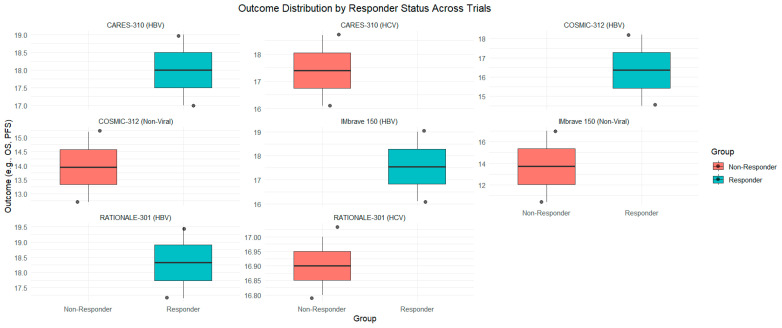
Comparison of OS and PFS between responders and non-responders across various trials.

Figure 7 illustrates the distribution of OS and PFS outcomes between responder and non-responder groups across different clinical trials, including CARES-310, COSMIC-312, IMbrave 150, and RATIONALE-301. The boxplots are segmented by trial and further divided into groups based on response status (responder vs. non-Responder) (Figure 7).

**Figure 8:**
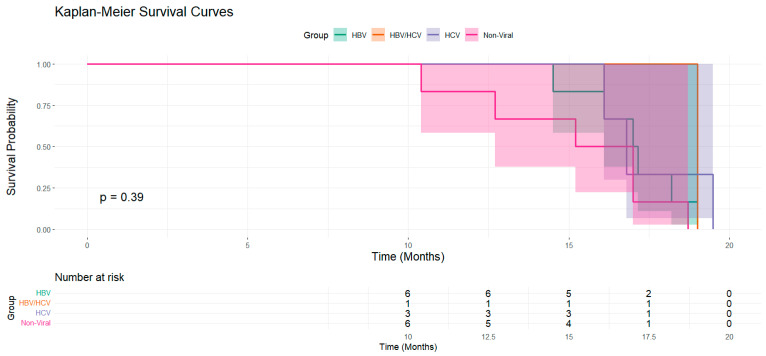
Kaplan–Meier survival curves comparing HBV, HBV/HCV, HCV, and non-viral groups.

Figure 8 displays Kaplan–Meier survival curves for patients categorized into four groups: HBV, HBV/HCV, HCV, and non-viral. The survival probability is plotted over time (in months), with the corresponding number at risk shown below the *x*-axis. The *p*-value of 0.39 indicates no statistically significant difference in survival between these groups over the follow-up period.

**Figure 9:**
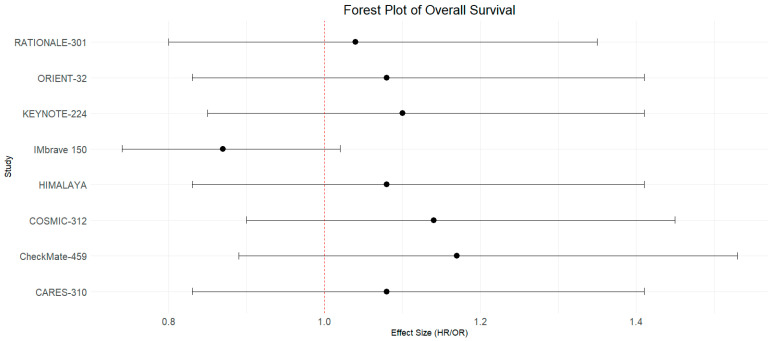
Forest plot of overall survival hazard ratios across clinical trials.

Figure 9 presents a forest plot summarizing the hazard ratios (HRs) for overall survival across multiple clinical trials, including RATIONALE-301, ORIENT-32, KEYNOTE-224, IMbrave 150, HIMALAYA, COSMIC-312, CheckMate-459, and CARES-310. The hazard ratios are plotted along with their corresponding 95% confidence intervals.

#### 3.2.4. Risk of Bias Findings

**Figure 10:**
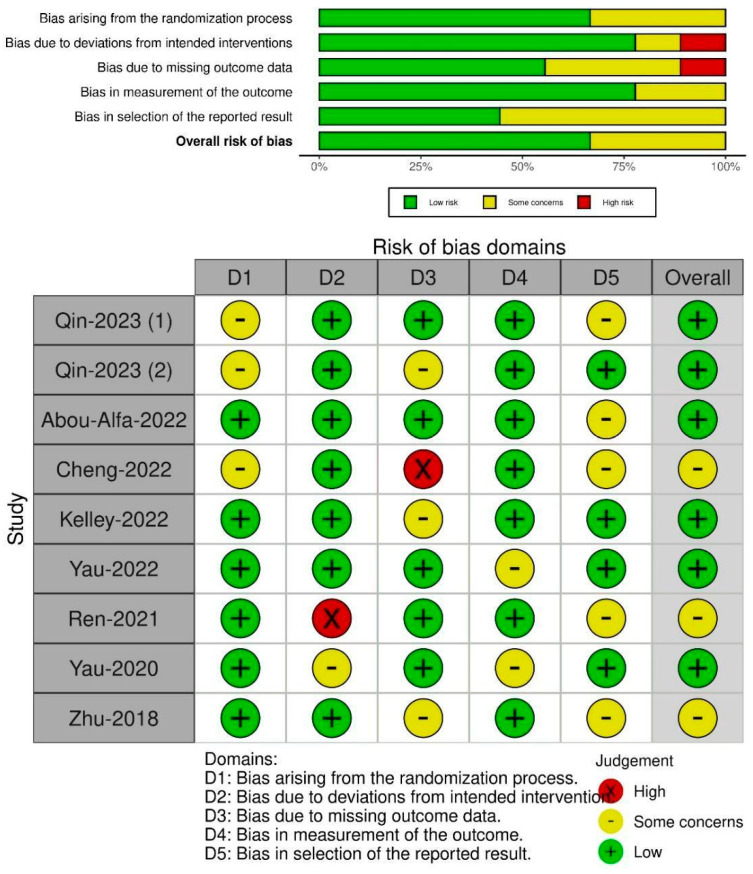
Summary trends of biases and study-by-study findings [34,35,36,37,38,39,40,41,42].

The summary traffic plot and study-by-study risk of bias findings are depicted in Figure 10.

Qin-2023 (RATIONALE-301) (1) [34] exhibited some concerns in Domain 1 (bias arising from the randomization process) and Domain 5 (bias in selection of the reported result) but had a low risk of bias in Domain 2 (bias due to deviations from intended interventions), Domain 3 (bias due to missing outcome data), and Domain 4 (bias in measurement of the outcome), leading to an overall low risk of bias.

Qin-2023 (CARES-310) (2) [35] had some concerns in Domain 1 (bias arising from the randomization process) and Domain 3 (bias due to missing outcome data), a low risk of bias in Domain 2 (bias due to deviations from intended interventions), Domain 4 (bias in measurement of the outcome), and Domain 5 (bias in selection of the reported result), with an overall low risk of bias.

Abou-Alfa-2022 (HIMALAYA trial) [36] had a low risk of bias in Domains 1 through 4, which are the same as above, with some concerns in Domain 5 (bias in selection of the reported result), resulting in an overall low risk of bias.

Cheng-2022 (IMbrave 150) [37] showed some concerns in Domain 1 (bias arising from the randomization process), a high risk of bias in Domain 3 (bias due to missing outcome data), some concerns in Domain 5 (bias in selection of the reported result), and a low risk of bias in Domain 2 (bias due to deviations from intended interventions) and Domain 4 (bias in measurement of the outcome), culminating in an overall judgment of some concerns.

Kelley-2022 (COSMIC-312) [38] had a low risk of bias in Domains 1, 2, 4, and 5, with some concerns in Domain 3 (bias due to missing outcome data), resulting in an overall low risk of bias.

Yau-2022 (CheckMate-459) [39] generally had a low risk of bias, with some concerns in Domain 4 (bias in measurement of the outcome), and low risk in all other domains, thus having an overall low risk of bias.

Ren-2021 (ORIENT-32) [40] had a high risk of bias in Domain 2 (bias due to deviations from intended interventions), some concerns in Domain 5 (bias in selection of the reported result), and a low risk of bias in the other domains, leading to an overall assessment of some concerns.

Yau-2020 (CheckMate 040) [41] had a low risk of bias in Domains 1, 3, and 5, with some concerns in Domain 2 (bias due to deviations from intended interventions) and Domain 4 (bias in measurement of the outcome), resulting in an overall low risk of bias.

Zhu-2018 (KEYNOTE-224 trial) [42] had a low risk of bias in Domains 1, 2, and 4 and some concerns in Domain 3 (bias due to missing outcome data) and Domain 5 (bias in selection of the reported result), which led to an overall judgment of some concerns.

## 4. Discussion

In recent years, the focus on immunotherapy, specifically ICIs, as a promising treatment avenue for HCC has led to an accumulation of clinical trial data exploring their efficacy across various patient demographics, including those differentiated by viral etiology. Our meta-analysis highlights significant differences in PFS among HCC patients based on the etiology of their disease. The extended PFS in HCV-infected patients compared to HBV-infected individuals suggests that the immunological landscape influenced by HCV may be more amenable to immunotherapy interventions. This observation could potentially be linked to HCV’s unique interaction with the host immune system, which may preserve immune responsiveness better than HBV, thereby enhancing the efficacy of ICIs. The notably shorter PFS in non-viral HCC patients points to a possibly different tumor biology, less responsive to immunotherapeutic strategies commonly effective in virally induced HCC. This trend underpins the necessity for developing alternative therapeutic approaches or combination therapies that can better target the unique pathways active in non-viral HCC.

The OS data reveal a key insight; while viral hepatitis-associated HCC patients generally benefit more from ICIs, the marginal difference in OS between HBV and HCV patients might indicate varying degrees of immune tolerance and tumor microenvironment factors influenced by the type of viral infection. HBV’s slightly superior OS could be attributable to either the nature of its oncogenic process or potentially more aggressive standard care protocols that are optimized for HBV-related liver cancer. The relatively lower OS in non-viral HCC patients further emphasizes the challenge of treating these cancers, possibly due to a lack of specific molecular targets that are present in viral hepatitis-related cancers. This disparity highlights the importance of precision medicine in HCC treatment, where understanding the molecular and biological underpinnings of each patient’s tumor could lead to more effective, tailored therapies.

In the context of the sensitivity and leave-one-out meta-analyses involving studies such as IMbrave 150, COSMIC-312, and CheckMate 040, along with trials assessing OS in HBV and HCV groups, it is crucial to highlight the effect sizes associated with specific interventions in HCC treatments. Notably, the removal of one arm from COSMIC-312 resulted in a marked increase in the effect size for OS, underscoring the profound influence and potential efficacy of the treatments studied in this particular trial. This change in effect size is pivotal as it provides insight into the specific contributions of individual trial components to overall outcomes and emphasizes the effectiveness of the therapies tested. In contrast, adjustments such as excluding arms from the HIMALAYA trial led to varying effect sizes with odds ratios shifting from 1.17 to 1.08, each accompanied by moderate to high heterogeneity. Similarly, the exclusion of other trials like IMbrave 150 consistently resulted in an odds ratio of 1.08, indicating a minimal impact on overall results. Meanwhile, the removal of trials like RATIONALE-301 and CARES-310 showed negligible influence on the meta-analytic conclusions, with odds ratios close to neutrality and non-significant overall effects.

These findings highlight the importance of individual study contributions to the pooled estimates and highlight the need for careful interpretation due to observed heterogeneity. Such detailed analyses are essential for validating the robustness and applicability of meta-analytic results, crucially aiding in the refinement of treatment protocols. This ensures that therapies are effectively tailored to meet the diverse needs of HCC patients across different viral etiologies, ultimately influencing clinical decision making and guiding future research directions.

Two notable meta-analyses conducted by Ding and colleagues until August 2020 [43] and Du and colleagues until February 2023 have provided key insights into this area [44]. Ding and colleagues led on a comprehensive search across several databases, concluding that the presence of viral infections (HBV or HCV) in HCC patients does not significantly influence the ORR to ICI therapies. Their analysis, involving 1520 patients across eight studies, revealed no substantial difference in ORR between patients with virally infected HCC and those without viral infections. Furthermore, they noted that while certain immune cells’ infiltration levels in the tumor microenvironment varied by etiology, this did not drastically affect the overall immune or stromal scores, suggesting a minimal impact of viral status on tumor immune microenvironment (TIME) remodeling. On the other hand, Du and colleagues’ findings, derived from a broad literature search including conference abstracts, indicated that ICIs significantly improve OS, PFS, and ORR across different etiological subgroups of HCC, with notably pronounced benefits in HBV-related HCC patients. Their meta-analysis of 5646 patients from eight studies highlighted that while ICI therapies benefited all studied subgroups, HBV patients demonstrated the most significant improvement, especially in terms of PFS.

Given these comprehensive analyses, the necessity for our current meta-analysis, conducted until 5 April 2024, emerges from the evolving landscape of HCC treatment and the continued expansion of clinical trial data. Our analysis sought to incorporate the latest studies post-February 2023, providing an updated synthesis of evidence in the era of rapidly advancing immunotherapy options. With the incremental accumulation of clinical trial outcomes and real-world evidence, there is a need to reassess and refine our understanding of the relationships between viral etiology, immune response, and the efficacy of ICIs in HCC treatment. Moreover, our meta-analysis delved deeper into the heterogeneity within the etiological subgroups and the potential differential impact of various ICI therapies, considering new therapeutic combinations and strategies that have emerged in the interim.

The projected increase in annual HCC incidence to over one million cases worldwide by 2025 highlights the urgency for refined treatment approaches tailored to at-risk patient groups, particularly considering the strong male predominance and the association of HCC with advancing age across all populations [45,46]. Risk factors such as HBV/HCV, metabolic dysfunction-associated steatohepatitis (MASH), and lifestyle-related factors such as heavy alcohol consumption and obesity highlight the complex etiology of HCC [47]. Our meta-analysis is a critical addition to the growing body of research focused on HCC, a disease that accounts for the majority of liver cancer cases and exhibits high morbidity and mortality rates [16,48,49,50,51].

### 4.1. Limitations

While this meta-analysis offers insights into the potential of immunotherapy in treating viral hepatitis-induced HCC, several limitations must be acknowledged. First, the inclusion of studies with differential designs, populations, and treatment regimens introduces variability, potentially impacting the generalizability of the findings. The predominance of phase 2 and phase 3 trials may reflect a certain level of selection bias, focusing on treatments that have already shown some promise, thereby potentially overlooking emerging therapies still in early-stage research. Additionally, the heterogeneity observed in some of our analyses, particularly regarding PFS in the viral vs. non-viral groups and within the HBV and HCV subgroups, suggests underlying differences in study populations, treatment modalities, or both, which could influence the outcomes. This heterogeneity, while accounted for through statistical methods, highlights the complexity of comparing immunotherapeutic responses across different etiological backgrounds.

Another limitation is the reliance on published data, which may be subject to publication bias, where studies with positive findings are more likely to be published. Furthermore, the meta-analysis did not systematically evaluate the quality of life and adverse effects associated with the immunotherapy treatments, aspects that are crucial for understanding the holistic impact of these therapies on patients’ lives. Lastly, the rapidly evolving landscape of immunotherapy research means that newer studies, potentially relevant to our analysis, could have been published after our search cut-off date.

Lastly, another limitation was the scarcity of data pertaining to active HBV or HCV disease status. The studies included did not specify whether HCV RNA became undetectable after anti-viral treatment. The prognosis differs between patients who are HCV RNA-negative and those with active hepatitis cases that are HCV RNA-positive.

### 4.2. Recommendations for Future Research and Clinical Practice

Based on the findings and limitations of this meta-analysis, several recommendations for future research and clinical practice are proposed. First, there is a need for additional, large-scale RCTs focusing on the efficacy of immunotherapy in HCC, particularly stratified by viral etiology and genotype, to better understand the relationships between viral infection status and treatment response. Second, beyond efficacy, future research should also systematically assess the quality of life, long-term survival, and adverse effects associated with immunotherapy treatments to provide an evaluation of their impact on patient care. Third, given the observed heterogeneity, future analyses should include more detailed subgroup analyses based on factors such as the presence of cirrhosis, degree of liver dysfunction, prior treatment history, and specific characteristics of the immunotherapy regimen (e.g., monotherapy vs. combination therapy). Fourth, to overcome the limitations associated with variability and potential publication bias, global collaboration and data sharing among researchers could facilitate more comprehensive and diverse analyses, improving the understanding of immunotherapy’s role in treating HCC. Fifth, clinicians ought to consider the viral etiology of HCC when selecting immunotherapy treatments, adjusting the approach to the individual’s specific disease characteristics and overall health status to optimize outcomes. Lastly, effective strategies for the early detection and management of immunotherapy-related adverse effects should be developed and integrated into treatment protocols to ensure patient safety and treatment continuity.

## 5. Conclusions

Our study reinforces the existing evidence that immunotherapy is particularly effective in viral hepatitis-associated HCC cases, with a significant impact observed in HBV-associated HCC. While previous studies have demonstrated better responses to ICIs in viral-related HCC compared to non-viral cases, our study provides a more comprehensive analysis, including the latest clinical trials and broader patient demographics. These findings underscore the importance of tailoring immunotherapy approaches based on the viral etiology of HCC and suggest that future research should focus on identifying molecular markers that can predict response to ICIs, enabling more personalized and effective treatment strategies for HCC patients. The results underscore the need to tailor immunotherapy strategies based on the viral etiology of HCC, emphasizing the importance of etiology-specific treatment planning. Notably, differences in progression-free and overall survival rates between HBV and HCV subgroups suggest that such tailored approaches could optimize outcomes. The comparable efficacy of ICIs across viral and non-viral HCC further supports their potential role as a standard component of care, regardless of underlying liver disease. Continued research is essential to keep pace with evolving HCC pathology and immunotherapeutic advancements. Future studies should aim to identify molecular markers and viral genotype for predicting ICI response, thereby enhancing the personalization and effectiveness of treatment strategies. By building on the knowledge foundation established here, we can significantly improve the prognosis and quality of life for patients with HCC, particularly those with a background of viral hepatitis.

## Figures and Tables

**Table 1:** Characteristics of the Included Studies [33,34,35,36,37,38,39,40,41].

Author-Year	Trial Name	Phase	N	Age (Median)	Male (%)	HBV Viral Load (IU/mL)	HBsAg	Prior Anti-Viral Therapy	HDV Coinfection	Intervention	Dosage	Follow-Up Duration	Outcomes: Group A—Viral Hepatitis	Outcomes: Group B—Non-Infected
Qin-2023 (1) [34]	RATIONALE-301	3	674	61 (23–86)	84.60%	NR	NR	NR	NR	Tislelizumab versus sorafenib	Tislelizumab, 200 mg IV every 3 weeks	4 years and 7 months	OS: HBV: 158/214 HCV: 26/46	OS: Non-Viral: 58/82
Qin-2023 (2) [35]	CARES-310	3	543	58 (46–56)	83%	NR	NR	NR	NR	Camrelizumab (PDL1) + rivoceranib (VEGFR) vs. sorafenib	Camrelizumab 200 mg Q2W and rivoceranib 250 mg PO QDS	PFS: 7.8 months (IQR: 4.1–10.6) OS: 14.5 months (IQR: 9.1–18.7)	PFS: HBV: 155/208 HCV: 9/22 OS: HBV: 90/208 HCV: 6/22	PFS: 25/42 OS: 15/42
Abou-Alfa-2022 [36]	HIMALAYA trial	3	1171	65 (22–86)	83.2	NR	Detectable	Yes	No	Tremelimumab + durvalumab versus sorafenib	Group A: tremelimumab (300 mg, one dose) plus durvalumab (1500 mg every 4 weeks) Group B: durvalumab (1500 mg every 4 weeks)	37.5 months	OS: Group A: HBV: 82/122 HCV: 73/110 Group B: HBV: 91/119 HCV: 73/107	OS: Group A: Non-Viral: 107/161 Group B: 116/163
Cheng-2022 [37]	IMbrave 150	3	501	NR	82.6	<500	Detectable	Yes	N/R	Atezolizumab + bevacizumab vs. sorafenib	1200 mg atezolizumab plus 15 mg/kg bevacizumab intravenously every 3 weeks or 400 mg sorafenib orally twice daily	10 months	RR: HBV: 50/158 HCV: 21/70 PFS: HBV: 129/164; 6.7 (5.4–9.5) HCV: 48/72; 8.8 (6.0–13.5) OS: HBV: 86/164; 19.0 (16.1-NE) HCV: 31/72; 24.6 (19.8-NE)	RR: 26/98 PFS: 80/100; 7.1 (5.6–9.6) OS: 63/100; 17.0 (11.7–22.8)
Kelley-2022 [38]	COSMIC-312	3	837	64 (58–70)	83	<500	Detectable	Yes	N/R	Atezolizumab (PDL1) + cabozantinib versus sorafenib	Cabozantinib 40 mg orally once daily plus atezolizumab 1200 mg intravenously every 3 weeks	Overall ITT: 13.3 months (IQR: 10.5–16) PFS ITT: 15.8 months (IQR: 14.5–17.2)	PFS: cabozantinib + atezolizumab: HBV: (52/74); 6.7 (5.6–8.3) HCV without HBV: (43/71); 7.9 (5.8–11) OS: cabozantinib + atezolizumab: HBV: (41/127); 18.2 (15.4-NE) HCV without HBV: (66/136); 13.6 (10.8–17)	PFS: cabozantinib + atezplizumab: (79/105); 5.8 (4.3–9.3) OS: cabozantinib + atezplizumab: (76/169); 15.2 (12.5-NE)
Yau-2022 [39]	CheckMate-459	3	743	65 (57–71)	85%	<100	Detectable	Yes	No	Nivolumab versus sorafenib	Nivolumab (240 mg intravenous every 2 weeks; n = 371) or sorafenib (400 mg oral twice daily; n = 372)	15.2 months (IQR: 5.7–28)	OS: nivolumab versus sorafenib, HBV: 16.1 vs. 10.4 months; HR 0.79 [95% CI, 0.59e1. 07] HCV: (17.5 vs. 12.7; HR, 0.72 [95% CI, 0.51e1. 02]	NR
Ren-2021 [40]	ORIENT-32	2–3	595	53 (21–82)	88%	less than 2000	Undetectable	N/R	N/R	Sintilimab + IBI305 versus sorafenib	Phase 2: IV sintilimab (200 mg every 3 weeks) plus IV IBI305 (15 mg/kg every 3 weeks) Phase 3: either sintilimab plus IBI305 (sintilimab–bevacizumab biosimilar group) or sorafenib (400 mg orally twice daily; sorafenib group), until disease progression or unacceptable toxicity	10 months (IQR: 8.5–11.7)	PFS: 179/359-HBV OS: 179/359-HBV	PFS: 12/21-non-infected OS: 12/21-non-infected
Yau-2020 [41]	CheckMate 040	2	148	60 (52–66)	81	<100	Detectable	Yes	No	Nivolumab versus sorafenib	Arm A: nivolumab 240 mg every 2 weeks Arm B: nivolumab 3 mg/kg plus ipilimumab 1 mg/kg, every 3 weeks (4 doses), followed by nivolumab 240 mg every 2 weeks Arm C: nivolumab 3 mg/kg every 2 weeks plus ipilimumab 1 mg/kg every 6 weeks	30.7 months (IQR: 29.9–34.7)	RR: Arm A: 9/28 (HBV), 2/7 (HCV) Arm B: 6/21 (HBV), 6/14 (HCV) Arm C: 8/26 (HBV), 5/12 (HCV) OS: HBV: Arm A: 22.8 (7.2-NE) (n = 50) Arm B: 12.1 (3.9–24.2) (n = 49) Arm C: 9.6 (6.0-NE) (n = 49) HCV: Arm A: 14.9 (0.7-NE) Arm B: 16.1 (6.5-NE) Arm C: 33.0 (3.1-NE)	RR: Arm A: 4/13 Arm B: 1/11 Arm C: 0/9 OS: Arm A: 22.2 (8.5-NE) Arm B: 11.8 (2.1–16.5) Arm C: 7.4 (0.9–14.5)
Zhu-2018 [42]	KEYNOTE-224 trial	2	104	68 (62–73)	83	<100	Undetectable	NR	NR	Pembrolizumab uncontrolled	200 mg pembrolizumab intravenously every 3 weeks for about 2 years	12.1 months	RR: 5/39 (HBC/HCV)	RR: Uninfected: 13/64

N: number of patients; NR: not reported; HDV: hepatitis D virus; Hbsag: hepatitis B surface antigen; OS: overall survival; PFS: progression-free survival. NR: Data are not reported separately for HBV/HCV groups.

## Data Availability

All data utilized for the purpose of this study are publicly available.
